# Case report: Anti-*N*-methyl-D-aspartate receptor antibody-associated autoimmunity triggered by primary central nervous system B-cell lymphoma

**DOI:** 10.3389/fneur.2022.1048953

**Published:** 2023-01-12

**Authors:** Yuki Yokota, Makoto Hara, Natsuki Oshita, Tomotaka Mizoguchi, Haruna Nishimaki, Hiroyuki Hao, Hideto Nakajima

**Affiliations:** ^1^Division of Neurology, Department of Medicine, Nihon University School of Medicine, Tokyo, Japan; ^2^Division of Oncologic Pathology, Department of Pathology and Microbiology, Nihon University School of Medicine, Tokyo, Japan; ^3^Division of Human Pathology, Department of Pathology and Microbiology, Nihon University School of Medicine, Tokyo, Japan

**Keywords:** primary central nervous system lymphoma, anti-NMDAR antibody, encephalitis, syndrome, diagnostic criteria

## Abstract

**Background:**

We herein detail our experience with a unique patient with a primary central nervous system (PCNS) B-cell lymphoma concomitant with anti-*N*-methyl-d-aspartate receptor (NMDAR) antibodies that satisfied the criteria of “probable anti-NMDAR encephalitis (ProNMDARE)” based on the Graus criteria 2016.

**Case presentation:**

A 73-year-old Japanese woman presented with acute pyrexia, agitation, and disturbance of consciousness. She gradually developed a reduction in speech frequency and truncal dystonia causing abnormal posture. Brain magnetic resonance imaging (MRI) demonstrated high-intensity lesions in the bilateral frontal lobes, and her cerebrospinal fluid revealed mild pleocytosis. She was diagnosed with acute encephalitis and treated with acyclovir and intravenous dexamethasone; however, no improvement was observed. She was transferred to our hospital 6 weeks after the onset of her symptoms, and anti-NMDAR antibodies were identified in her cerebrospinal fluid through indirect immunolabeling with rat brain frozen sections and cell-based assays with NR1/NR2 transfected HEK cells. Follow-up MRI showed enlargement of the lesions in the right frontal lobe with gadolinium enhancement, suggesting a brain tumor. Stereotactic surgery was implemented, with subsequent pathological examination revealing that the tumor was consistent with diffuse large B-cell lymphoma (DLBCL) without evidence of systemic satellite lesions. Stereotactic irradiative therapies were then added to her treatment regimen, which partly improved her neurological symptoms with only mild cognitive dysfunction still remaining. A decrease in anti-NMDAR antibody titer was also confirmed after immunotherapy and tumor removal.

**Conclusions:**

We herein report our experience with a novel case of PCNS-DLBCL masquerading as anti-NMDAR encephalitis that satisfied the diagnostic criteria of “proNMDARE.” Treatment, including tumor removal, ameliorated disease severity and antibody titers of the patient. Our findings suggest that anti-NMDAR antibody-associated autoimmunity can be triggered by PCNS B-cell tumors, although primary brain tumors need to be excluded before establishing a diagnosis of autoimmune encephalitis.

## Introduction

Anti-*N*-methyl-d-aspartate receptor (NMDAR) encephalitis is a well-characterized autoimmune encephalitis (AE) associated with immunoglobulin (Ig) G against the GluN1 subunit of the NMDAR (1). In 2016, Graus et al. (Graus criteria) proposed a syndrome-based diagnostic approach to AE that included NMDAR encephalitis (NMDARE) (2). The Graus criteria for probable NMDARE (ProNMDARE) comprises major groups of neuropsychiatric symptoms, laboratory study results, and reasonable exclusion of other disorders, whereas definite NMDARE requires the detection of the NMDAR IgG mainly in the CSF. In addition, associated teratoma, especially a systemic teratoma, is an important criterion for the diagnosis of NMDARE. Recently, primary central nervous system (PCNS) tumors, such as glioblastoma (3) and lymphomatosis cerebri (4), have been reported to occur concomitantly with NMDAR antibodies; however, the clinical significance of the antibodies associated with primary brain tumors has yet to be sufficiently discussed given that brain tumors need to be excluded before establishing a diagnosis of AE according to the criteria (2).

We herein report a patient with PCNS diffuse large B-cell lymphoma (DLBCL) concomitant with NMDAR antibodies in the CSF who developed acute encephalitis satisfying the diagnostic criteria of “proNMDARE.”

## Case presentation

A 73-year-old Japanese woman was admitted to another hospital for pyrexia, agitation, and mild disturbance of consciousness. She was initially diagnosed with a urinary tract infection, for which antibiotics were administered. However, her neurological condition gradually deteriorated. Brain MRI showed high-intensity lesions in the right temporal and bilateral frontal lobes on fluid-attenuated inversion recovery (FLAIR) images. CSF examination showed mild pleocytosis (47/mm^3^, monocyte predominant). She was diagnosed with acute encephalitis and was started intravenous acyclovir and intravenous dexamethasone.

**Figure 1:**
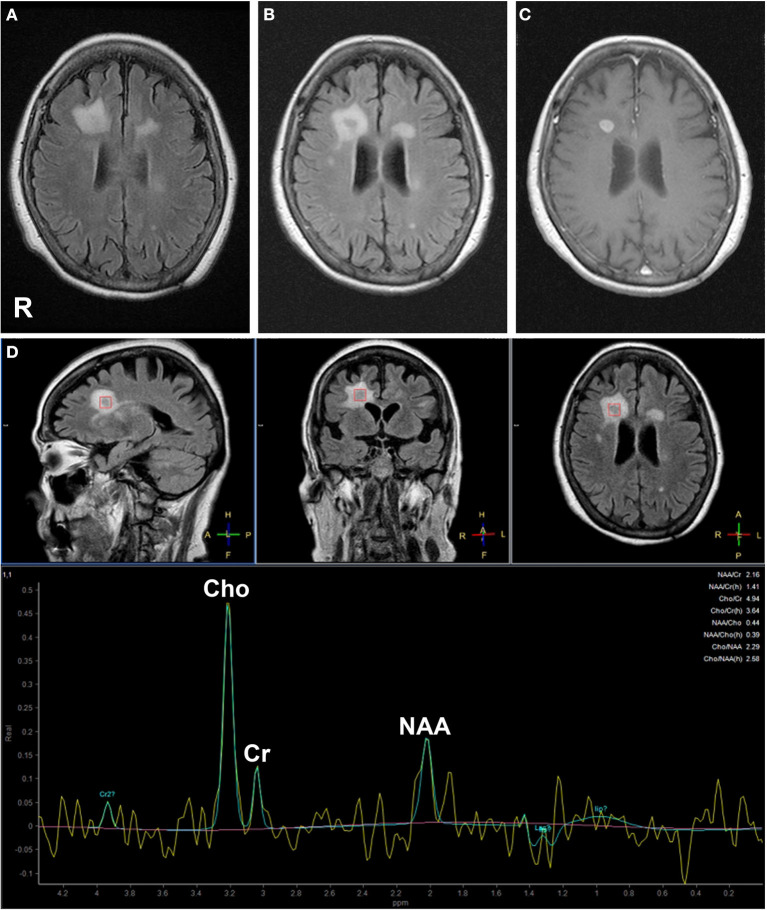
Brain magnetic resonance (MR) fluid-attenuated inversion recovery (FLAIR) images **(A, B)**, gadolinium (Gd)-enhanced T1-weighted image **(C)**, and MR spectroscopy **(D)** findings. Brain MRI FLAIR 7 days before admission revealed high-intensity lesions in the bilateral frontal lobes **(A)**. MRI FLAIR **(B)** and Gd-enhanced T1-weighted image **(C)** on admission revealed that high-intensity area in the right frontal lobe on MRI FLAIR showed round shaped on Gd enhancement. MR spectroscopy demonstrated an increase in choline/creatine ratio and decrease in N-acetyl aspartate levels in the Gd-enhanced lesion **(D)**.

She was transferred to our hospital 6 weeks after the onset of her symptoms due to persistent disease despite treatment. Her medical history included hypertension. She had no family history of neuromuscular diseases. On admission, the patient had a height and weight of 154.0 cm and 56 kg, respectively. On physical examination, her body temperature was 37.4°C. Chest auscultation revealed normal respiratory sounds and a normal heart rate with no murmur. Neurological examination showed mild disturbance of consciousness: Glasgow Coma Scale (GCS) 13 (E4 V4 M5) with disorientation and agitation. She was able to answer some simple questions but showed a remarkable reduction in speech frequency. She presented with cervical and truncal dystonia causing abnormal posture. She did not experience seizures. Autonomic dysfunction and central hypoventilation were unremarkable. Electroencephalography (EEG) revealed a generalized slowness in activity (5–7 Hz) without epileptiform discharges. A cerebrospinal fluid (CSF) sample included 5 white blood cells/mm^3^, 18 mg/dl of total protein, and 77 mg/dl of glucose (149 mg/dl of serum glucose). Her IgG index (0.50) was within normal range (cut-off < 0.67). Her CSF sample came back positive for oligoclonal bands. Brain MRI revealed high-intensity lesions in the right temporal and bilateral frontal lobes on FLAIR images, with the right frontal lesion demonstrating gadolinium (Gd) enhancement, which can be considered to indicate CNS inflammation or a high-grade brain tumor. Magnetic resonance spectroscopy of the right frontal lesion revealed an increase in the choline/creatine ratio and a normal N-acetyl aspartate level (Figure 1). Whole-body contrast-enhanced computed tomography showed no associated tumor and swelling of systemic lymph nodes. In addition, ^67^Ga scintigraphy showed no abnormal uptake in the systemic organs and lymph nodes (Supplementary Figure 1).

**Figure 2:**
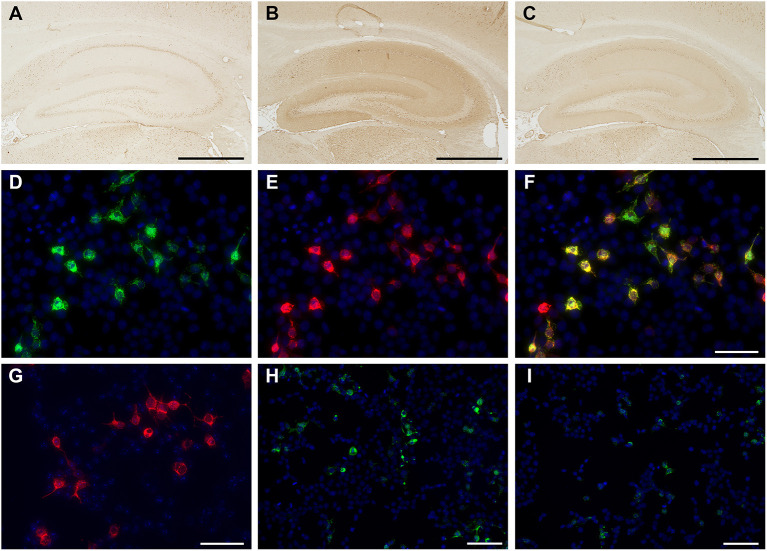
Immunolabeling of the patient's cerebrospinal fluid (CSF) sample with in-house tissue-based assay (TBA) **(A–C)** and cell-based assay (CBA) **(D–I)**. Control CSF did not react with in-house TBA **(A)**, whereas the initial patient's CSF immunolabeled neuropil on the rat hippocampus in the TBA **(B)**. The patient's CSF showed reactivity [green, **(D)**], with human embryonic kidney (HEK) cells expressing the *N*-methyl-d-aspartate receptor (NMDAR). The commercial antibody against NMDAR1 (clone EPR2480Y, ab68144, Abcam) [red, **(E)**] colocalizes with that of the patient's CSF [yellow, **(F)**], whereas a control CSF is negative **(G)**. Anti-NMDAR antibody titers (1:32) were confirmed using the fluorescent reactivity of CBA on the initial patient's CSF **(H)**. Following tumor removal and whole-brain irradiation therapy, immunosignals in TBA **(C)** and CBA decreased, as did antibody titers of anti-NMDAR antibody (titers 1:4) **(I)**. Nuclei were counterstained with 4′, 6-diamino-2-phenylindole. Bars = 1000 μm **(A–C)**, 50 μm **(D–G)**, and 100 μm **(H, I)**.

In-house screening for anti-neuronal antibodies (5, 6) was performed using the patient's CSF and sera samples *via* indirect immunohistochemistry with frozen sections of rat brain. Patient's CSF immunolabeled neuropil of the rat hippocampus (Figure 2) and granular cell layers in the cerebellum (Supplementary Figure 2), which was consistent with the immunolabeling pattern of anti-NMDAR antibodies. In contrast, the patient's serum did not immunolabel the rat brain section. A cell-based assay with NR1/NR2-transfected HEK293 cells (BIOCHIP, Euroimmun, Labor Berlin) confirmed anti-NMDAR antibodies in the CSF alone (Figure 2, antibody titers were 1:32). No onconeural antibodies that included amphiphysin, Hu, Yo, CV2, Ri, Ma2/Ta, recoverin, Tr, GAD65, and others measured *via* line blot (EUROLINE, Euroimmun, Lübeck, Germany) were detected (details regarding autoantibody detection using in-house and commercially available assays are described in the Supplementary methods). Furthermore, autoantibodies associated with other AE were not detected, including Bickerstaff's brainstem encephalitis (anti-GQ1b antibody in the serum), Hashimoto's encephalitis (antibodies against thyroid peroxidase and thyroglobulin in the serum), and demyelinating disorders of the CNS (antibodies against anti-myelin oligodendrocyte and aquaporin-4 in both the CSF and serum).

**Figure 3:**
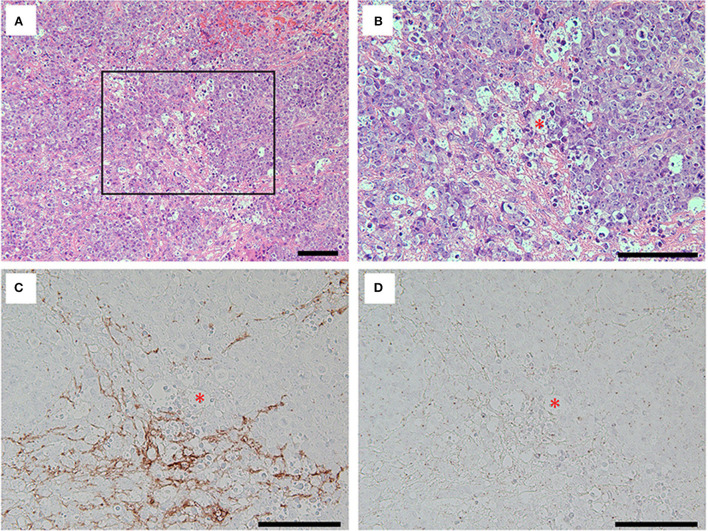
Hematoxylin and eosin (H&E) staining **(A, B)** and immunolabeling with the antibodies against grail fibrillary acidic protein (GFAP) (clone G-A-5; Sigma, MO, US) **(C)** and NR1 **(D)** for the tumor cells. Note that H&E staining of the removed brain specimens showed foci of highly proliferative atypical cells interposed with basophilic matrix **(A)**. The tumor cells with irregular nuclei and frequent mitoses [**(B)**, inset in **(A)**] were not immunolabeled with commercial antibodies against GFAP **(C)** nor NR1 subunit of NMDAR **(D)**, while the basophilic matrix on H&E staining was immunolabeled with commercial antibody against GFAP **(C)**. Asterisks in red **(B–D)** indicate the same blood vessel, and all bars = 100 μm.

The Gd-enhanced lesion accompanied by swelling of right frontal robe was required to rule out a high-grade brain tumor; thus, craniotomy was performed for tumor resection on day 15 of the hospitalization. Histopathological analyses showed a dense proliferation of atypical cells with large and irregular nuclei on hematoxylin-eosin (H&E) staining. Tumor cells were positive for CD20 (clone L26; Agilent Technologies, CA, US) and CD79a (clone JCB117; Agilent Technologies, CA, US) suggesting B-cell lymphoma with high Ki-67 (clone MIB-1; Agilent Technologies, CA, US) expression (90%). Immunological profiles of the tumor were consistent with primary central nervous system diffuse large B-cell lymphoma (PCNS-DLBCL) (Supplementary Figure 3). In addition, immunolabeling with biopsied brain sections using anti-NR1 antibodies (clone EPR23397-66; Abcam, Cambridge, UK) revealed that the tumor cells did not express the NR1 subunit of NMDAR (Figure 3).

After tumor removal, the patient underwent whole-brain stereotactic irradiation therapies (a total of 30 Gy). The antibody titers of anti-NMDAR antibodies in the CSF decreased as the volume of brain tumor reduced after surgical and irradiative therapies (Figure 2, titers were 1:4). Her consciousness and mental condition partially improved; however, she was discharged to another hospital for rehabilitation and long-term care due to limitations in performing activities of daily living. The clinical course of the patient is summarized in Supplementary Figure 4. Written informed consent was obtained from the patient and patient's kin for the publication of any potentially identifiable images or data included in this article.

## Discussion

We herein describe a case of PCNS-DLBCL that clinically manifested as acute encephalitis associated with anti-NMDAR antibody. Fortunately, a combination of tumor removal and irradiative therapies was able to decreased her antibody titers. The present case satisfied the criteria for proNMDARE (four of the six major symptoms and abnormal EEG finding) proposed by Graus et al. (2). In-house screening of the antibodies against neuronal surface antigens (5, 6), which included anti-NMDAR antibodies, could help with the early identification of antibodies in our patient. After testing positive for anti-NMDAR IgG in her CSF, she also satisfied the diagnostic criteria for definite NMDARE. However, systemic tumor analyses identified a DLBCL isolated in the CNS without radiological involvement of the systemic lymph nodes and organs. According to the aforementioned criteria (2), brain tumors are one of the diseases that need to be excluded before establishing a diagnosis of AE. Thus, we eventually diagnosed the patient with PCNS-DLBCL masquerading as NMDARE.

Recent studies have suggested the association between some types of neuronal surface antibodies (NSA) and hematologic neoplasms (7–10). For instance, Hodgkin lymphoma was associated with anti-NMDAR antibody (7) and mGluR5 antibody (8), whereas non-Hodgkin's lymphoma was associated with anti-DPPX antibody (9). Interestingly, Thomas et al. recently described a case with PCNS lymphoma mimicking anti-LGI1 limbic encephalitis. They mentioned that the case showed low serum anti-LGI1 antibody titers not detected in the CSF (10). In these reports (7–10), the patients were considered to have exhibited paraneoplastic syndrome considering that amelioration (8, 9) or deterioration (10) of the neurological syndrome was identified depending on the tumor condition. In addition, reports have also shown that the expression of the neuronal surface antigens was not confirmed on the surface of neoplastic lymphocytes (11, 12). In our case, the neurological syndrome partly improved, with only mild cognitive dysfunction remaining after the tumor removal following irradiative therapies. Furthermore, the tumor cells did not express NR1 subunits of NMDAR (Figure 3D), and no other associated tumors outside of the CNS (e.g., ovarian teratoma) were detected. Considering these facts and aforementioned evidence, it is likely that the PCNS lymphoma in our case induced paraneoplastic NMDARE. However, it seems unreasonable that an ectopic expression of NMDAR on the tumor cells could yield anti-NMDAR antibodies as observed in patients with systemic teratomas and other associated tumors (13, 14).

Conversely, it is wellknown that anti-NMDAR antibody titers in the CSF corresponded to the severity of the disorder and that most patients experienced a decrease in the titers following treatment that included tumor removal (for paraneoplastic cases) and intensive immunotherapies (15). The serial CSF antibody titers throughout the disease course in cases reported to be associated with NSA accompanied by hematologic neoplasms have not been thoroughly discussed (4, 7). Interestingly, in our case, a decrease in the antibody titers in the CSF was confirmed after treatments for the PCNS lymphoma. This finding also suggests that the neurological syndrome of the present case behaved as a paraneoplastic NMDARE.

Thereafter, we reviewed previous cases with primary brain tumors concomitant with anti-NMDAR antibodies (3, 4, 16) (Table 1). Fujii et al. (3) reported a 54-year-old woman with glioblastoma and ovarian teratoma who presented with complex partial seizures and impaired consciousness. Lu et al. (16) also reported a 67-year-old man with a brain astrocytoma who developed partial seizures. Interestingly, encephalitis worsened as the tumor progressed, although alterations in antibody titers were not examined. Mariotto et al. (4) reported a 54-year-old man who developed various types of cognitive impairment and was treated with plasma pheresis and cyclophosphamide. She was diagnosed with lymphomatosis cerebri following an autopsy. All previous cases developed limited syndrome (3, 4, 16) of NMDARE based on the Graus criteria (2). In contrast, our case developed typical anti-NMDAR syndrome and satisfied the criteria for “proNMDARE” (4 of the 6 major symptoms and abnormal EEG finding) (2). Moreover, in-house screening immediately detected anti-NMDAR antibody in the CSF. The prognosis of the cases depended on the malignancy potential of the associated brain tumors, which was relatively poor compared to a typical NMDARE (17). In our case, anti-NMDAR syndrome partly improved; however, she could not return to baseline after having been debilitated by PCNS-DLBCL, including a series of treatments for the tumors.

**Table 1:** Literature review of brain tumors concomitant with anti-NMDAR antibodies.

	**Fujii et al. (3)**	**Mariotto et al. (4)**	**Lu et al. (16)**	**Present case**
Age/sex	54/F	54/M	67/M	73/F
Symptoms	Complex partial seizure with impaired consciousness	Depression and emotional lability, left hand dystonia, attention deficit, anosognosia, visuospatial impairment, constructional apraxia	Left limb convulsions without loss of consciousness	Cognitive dysfunction, reduction in speech frequency, abnormal postures, decreased level of consciousness
Associated tumor	Glioblastoma, Ovarian teratoma	Lymphomatosis cerebri	Brain astrocytoma	Primary CNS B-cell lymphoma
Major groups of symptoms on ProNMDARE	Seizures	Abnormal (psychiatric) behavior/cognitive dysfunction, Movement disorder	Seizures	Cognitive impairment, speech dysfunction, movement disorders, decreased level of consciousness
Main syndrome	Limited features	Limited features	Limited features	Anti-NMDAR syndrome
CSF cell count (mm^3^)/TP (mg/dl)	2/30	Within normal limits	4/45	47/23
CSF NMDAR Abs	+	+	+	+
EEG findings	Focal slow activity, epileptic activity	Non-specific EEG changes	Slight abnormality	Diffuse slow activity
Treatments	Tumor resection (ovarian teratoma, glioblastoma), radiotherapy/chemotherapy (glioblastoma)	Plasma pheresis, cyclophosphamide	Dexamethasone, methylprednisolone, immunoglobulin, azathioprine, radiotherapy/chemotherapy	Dexamethasone, tumor resection/radiotherapy
mRS peak/latest	ND	6/6	6/6	5/5
Follow-up period, months	6	24	5	4

Abs, antibodies; CNS, central nervous system; CSF, cerebrospinal fluid; EEG, electroencephalogram; F, female; M, male; mRS, modified Rankin scale; NMDAR, anti-N-methyl-d-aspartate receptor; ND, not described; ProNMDARE, probable NMDAR encephalitis on Graus criteria.

Regarding the mechanism for the association between brain tumors and production of autoantibodies, Mariotto et al. (4) suggested that dysregulated lymphoma cells or exposure to specific antigens caused by brain damage yielded autoantibodies. The aforementioned mechanism is similar to the mechanism proposed in NMDARE subsequent to herpes simplex encephalitis (post-HSE) (18, 19). Namely, viral-induced neuronal lysis of the CNS can induce autoimmune reaction to produce the NSAs that include anti-NMDAR antibodies in the patients with post-HSE (19–23). We challengingly speculated that the production of anti-NMDAR antibodies in the present case was mainly associated with antigen exposure of the damaged brain caused by tumor progression given that concomitant PCNS-DLBCL aggressively infiltrated the right frontal lobe with higher expression rate of Ki-67 (Supplementary Figure 3D), a marker for determining growth fraction (24). Further investigation is required to elucidate the precise mechanisms of NSA production induced by the brain tumors.

In conclusion, we herein report a unique case involving PCNS-DLBCL who developed acute encephalitis satisfying the diagnostic criteria for “probable NMDARE.” The present case indicated that anti-NMDAR antibody-associated autoimmunity can be triggered by PCNS B-cell tumors, which are involved in anti-NMDAR syndrome presenting as a paraneoplastic NMDARE. Furthermore, brain tumor assessment even for patients suspected of AE can help prevent delays in the induction of appropriate treatment.

## Data availability statement

The original contributions presented in the study are included in the article/Supplementary material, further inquiries can be directed to the corresponding author.

## Ethics statement

Written informed consent was obtained from the individual(s) and/or minor(s)' legal guardian/next of kin for the publication of any potentially identifiable images or data included in this article.

## Author contributions

YY and MH drafted the manuscript. YY, MH, NO, TM, and HNa prepared patient's clinical data. HNi and HH performed the histological examination. MH, HH, and HNa supervised this study. All authors analyzed and interpreted the patient data and revised the manuscript for intellectual content. All authors approved the final manuscript.
