# Supplementary material for: Case report: Anti-N-methyl-D-aspartate receptor antibody-associated autoimmunity triggered by primary central nervous system B-cell lymphoma
Source: Front Neurol. 2023 Jan 12;13:1048953. doi: 10.3389/fneur.2022.1048953 (PMC9878211; doi:10.3389/fneur.2022.1048953)
Supplement: Supplementary file 1 [file Data_Sheet_1.pdf]

Supplementary Material

1 Supplementary Methods

1.1 In-house tissue-based assay (TBA) for the screening of neuronal surface antibodies (NSAs) and onconeural antibodies

A series of NSAs [e.g., antibodies against *N*-methyl-D-aspartate receptor (NMDAR), leucine-rich glioma-inactivated 1 (LGI1), contactin-associated protein-like 2 (Caspr2), dipeptidyl-peptidase-like protein 6 (DPPX), and immunoglobulin-like cell adhesion molecule 5 (IgLON5)] and onconeural antibodies (e.g., ANNA1, Yo, Ri, Ma, and CV2) in the patient's CSF and serum samples were screened using tissue-based assay (TBA).

TBA, which involved immunohistochemical analyses of rat brain tissue, was implemented as reported (1, 2). Briefly, adult female Wistar rats were sacrificed without perfusion, and the brain was removed and fixed in 4% paraformaldehyde for 1 h at 4 °C, cryoprotected in 40% sucrose for 48 h, embedded in freezing compound media, and snap frozen in isopentane chilled with liquid nitrogen. Thereafter, 6-μm-thick tissue sections were sequentially incubated with 0.3% H_2O_2 for 15 min, 5% goat serum for 1 h, and patient and control CSF (1:2) or sera (1:200) at 4 °C overnight. After incubating with biotinylated secondary antibodies against human IgG (1:2000, BA-3000, Vector), reactivity was developed using the avidin–biotin–peroxidase method. The results of the assay were independently evaluated by two experts (MH and HN) familiar with the immunohistochemical technique. Samples deemed "positive" were subsequently examined using the confirmation tests described below.

1.2 Confirmation tests of NSAs and onconeural antibodies with commercially available tests

To determine specific neuronal antigens, subsequent confirmation tests using commercially available cell-based assay (CBA) for seven neuronal surface antigens (NMDAR, α-amino-3-hydroxy-5-methyl-4-isoxazolepropionic acid receptor, LGI1, Caspr2, gamma-aminobutyric acid receptor type B, DPPX, and IgLON5) (BIOCHIP, Euroimmun, performed by Labor Berlin) and commercially available line blot assays for 12 onconeural antigens (EUROLINE, Euroimmun, Lübeck, Germany) were performed (2).

2 Supplementary Figures

2.1 Supplementary Figure 1. ^{67}Ga scintigraphy findings

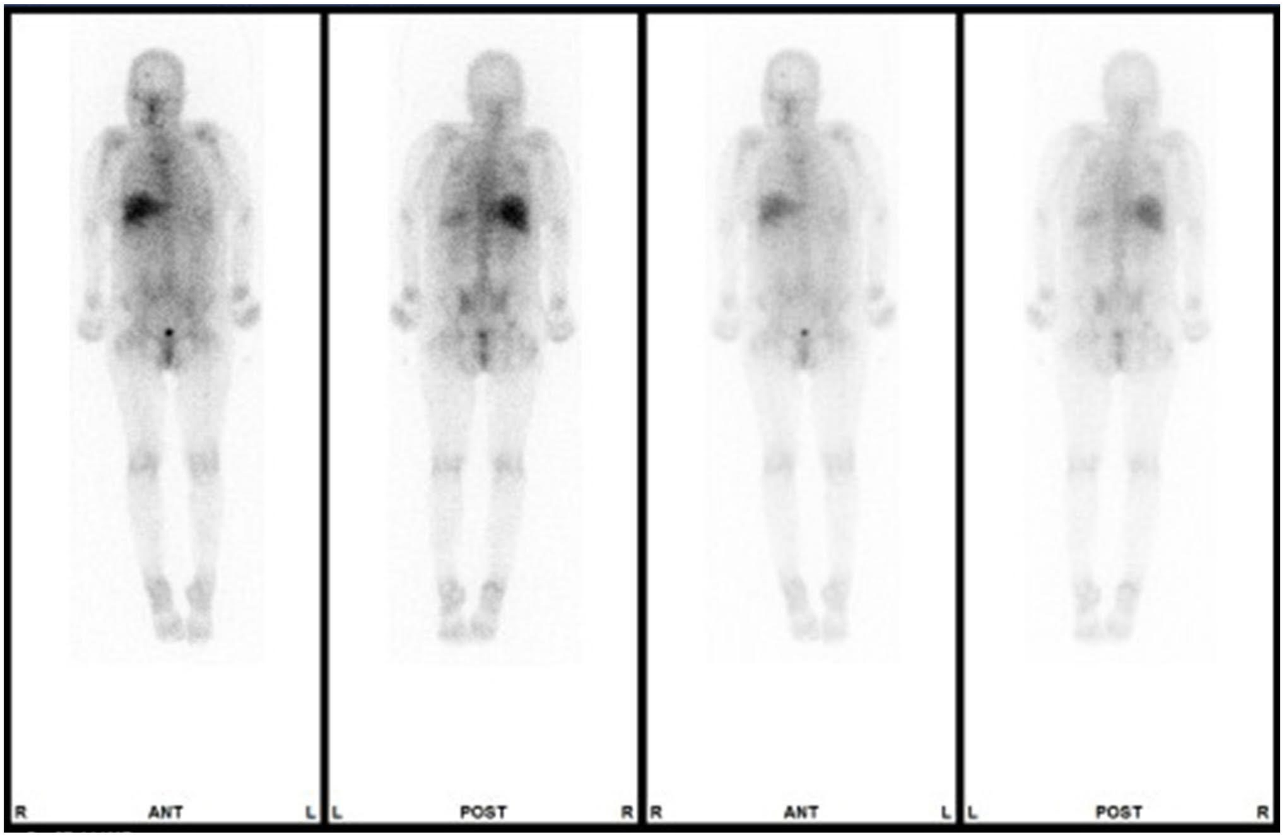

^{67}Ga scintigraphy findings for malignancy screening that includes systemic organs and lymph nodes. ^{67}Ga scintigraphy showed no abnormal uptake throughout the whole body.

2.2 Supplementary Figure 2. In-house indirect immunohistochemistry (IHC) using rat cerebellar sections permeabilized with 0.5% Triton X-100 for screening of autoantibodies against paraneoplastic intracellular antigens

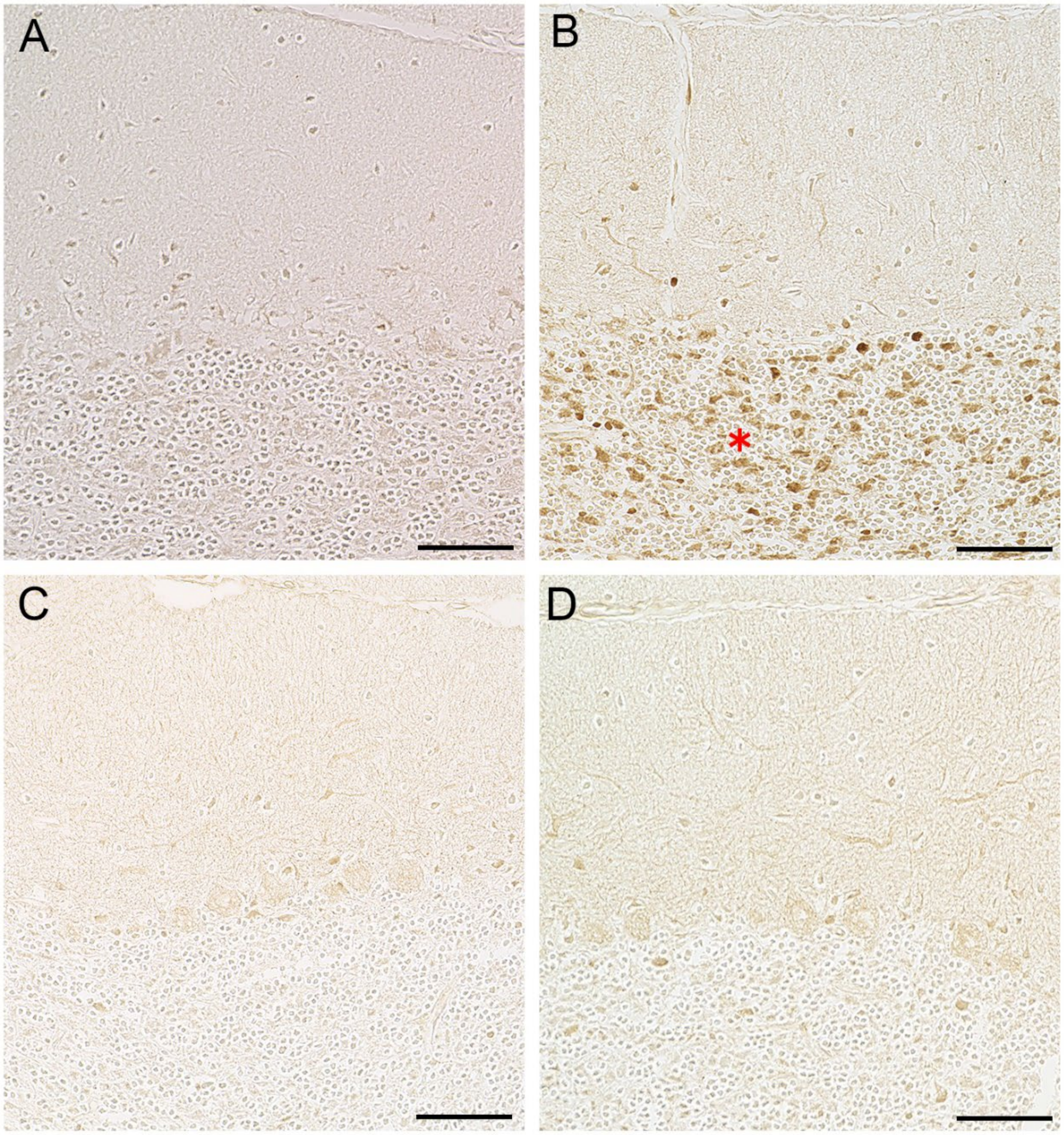

IHC was performed on rat cerebellar sections for control or patient CSF (A, B) and serum samples (C, D). Note that control CSF (A) did not label the rat cerebellum, while the patient’s CSF (B) labeled the synapses of granular cell layers (B, asterisk) in the cerebellum, which was attributed to anti-NMDAR antibodies, as reported (3, 4). The rat cerebellum was not labeled by either the control (C) or the patient's (D) serum samples. In-house IHC results revealed no evidence of autoantibodies against paraneoplastic intracellular antigens in the patient’s CSF or serum samples. All bars indicate 50 μm.

2.3 Supplementary Figure 3. Hematoxylin and eosin (H&E) staining and immunohistochemistry (IHC) of the removed tumor

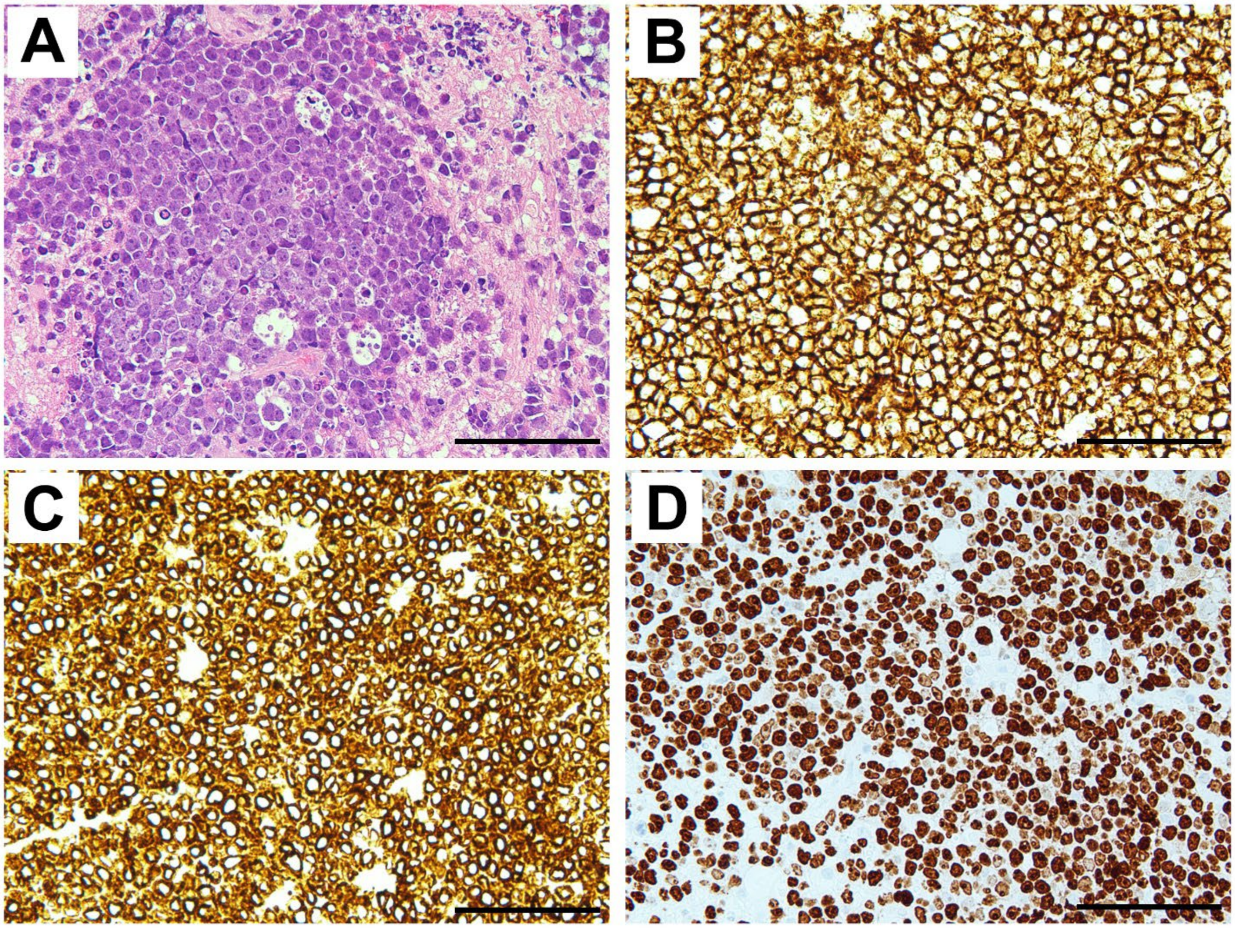

H&E staining (panel A) and IHC (panels B–D) of the excised brain tumor. H&E staining of the excised brain specimens revealed a dense proliferation of atypical cells with large and irregular nuclei (A). Tumor cells were immunolabeled with CD20 (B) and CD79a (C), which is consistent with B-cell lymphoma and shows high Ki-67 expression rate (approximately 90%) (D). All bars indicate 100 μm.

2.4 Supplementary Figure 4. Summary of the patient's clinical course

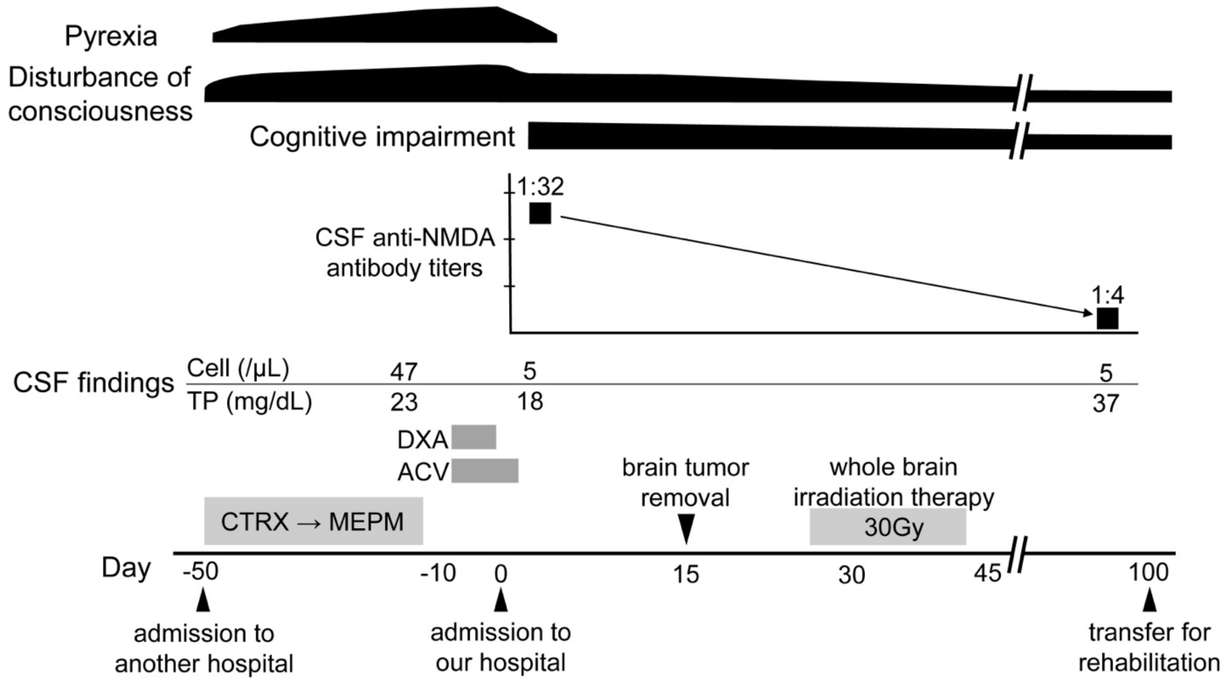

The patient was admitted to another hospital for acute onset of pyrexia and mild disturbance of consciousness, during which antibiotics were administered for urinary tract infection. However, her symptoms worsened and gradually involved cognitive impairment concomitant with a reduction in frequency of speech and truncal dystonia causing an abnormal posture. Her cerebrospinal fluid (CSF) test revealed mild pleocytosis. Thus, she was diagnosed with acute encephalitis and treated with acyclovir and intravenous dexamethasone to no avail. She was then transferred to our hospital 6 weeks after the onset of her symptoms. Anti-*N*-methyl-D-aspartate receptor (NMDAR) antibody was identified in her CSF with indirect immunolabeling with rat brain frozen sections and cell-based assays with NR1/NR2-transfected HEK cells (titer 1:32). Brain MRI showed a gadolinium-enhanced lesion in the right frontal area, suggesting a brain tumor. Stereotactic surgery was performed, and subsequent pathological examination suggested a tumor consistent with diffuse large B-cell lymphoma (DLBCL) without evidence of systemic satellite lesions. Stereotactic irradiative therapies were added to her regimen (a total of 30 Gy), and her neurological symptoms partly improved, although mild cognitive dysfunction remained. A decrease in anti-NMDAR antibody titer (titer 1:4) was also confirmed after immunotherapy and tumor removal. Her consciousness and mental condition partially improved; however, on her 100th day of hospitalization, she was discharged to another hospital for rehabilitation and long-term care due to limitations in performing activities of daily living.

3. Supplementary references

1. Hara M, Martinez-Hernandez E, Ariño H, Armangué T, Spatola M, Petit-Pedrol M, et al. Clinical and pathogenic significance of IgG, IgA, and IgM antibodies against the NMDA receptor. *Neurology* (2018) 90(16):e1386-e94. doi:10.1212/wnl.0000000000005329

2. Mizoguchi T, Hara M, Hirose S, Nakajima H. Novel qEEG Biomarker to Distinguish Anti-NMDAR Encephalitis From Other Types of Autoimmune Encephalitis. *Front Immunol* (2022) 13:845272. doi:10.3389/fimmu.2022.845272

3. Budhram A, Dubey D, Sechi E, Flanagan EP, Yang L, Bhayana V, et al. Neural Antibody Testing in Patients with Suspected Autoimmune Encephalitis. Clin Chem (2020) 66(12):1496-1509. doi:10.1093/clinchem/hvaa254.

4. Landa J, Guasp M, Míguez-Cabello F, Guimarães J, Mishima T, Oda F, et al. Encephalitis with Autoantibodies against the Glutamate Kainate Receptors GluK2. Ann neurol (2021) 90(1):101-117. doi: 10.1002/ana.26098.
